# Integrated metabonomic–proteomic studies on blood enrichment effects of *Angelica sinensis* on a blood deficiency mice model

**DOI:** 10.1080/13880209.2017.1281969

**Published:** 2017-01-31

**Authors:** Yongli Hua, Wangling Yao, Peng Ji, Yanming Wei

**Affiliations:** College of Veterinary Medicine, Gansu Agricultural University, Lanzhou, Gansu Province, People’s Republic of China

**Keywords:** GC-MS, 2D gel electrophoresis, hematopoietic activities

## Abstract

**Context:***Angelica sinensis* (Oliv.) Diels (Umbelliferae) (AS) is a well-known Traditional Chinese Medicine (TCM) that enriches and regulates the blood.

**Objective:** An integrated metabonomic and proteomic method was developed and applied to study the blood enrichment effects and mechanisms of AS on blood deficiency (BD) mouse model.

**Materials and methods:** Forty mice were randomly divided into the control, BD, High-dose of AS (ASH), Middle-dose of AS (ASM), and Low-dose of AS (ASL) groups. BD model mice were established by injecting *N*-acetylphenylhydrazine (APH) and cyclophosphamide (CTX) (ip). The aqueous extract of AS was administered at three dose of 20, 10, or 5 g/kg b. wt. orally for 7 consecutive days before/after APH and CTX administration. Gas chromatography–mass spectrometry (GC-MS) combined with pattern recognition method and 2D gel electrophoresis (2-DE) proteomics were performed in this study to discover the underlying hematopoietic regulation mechanisms of AS on BD mouse model.

**Results:** Unlike in the control group, the HSP90 and arginase levels increased significantly (*p <* 0.05) in the BD group, but the levels of carbonic anhydrase, GAPDH, catalase, fibrinogen, GSTP, carboxylesterase and hem binding protein in the BD group decreased significantly (*p* < 0.05). Unlike the levels in the BD group, the levels of these biomarkers were regulated to a normal state near the control group in the ASM group. Unlike in the control group, l-alanine, arachidonic acid, l-valine, octadecanoic acid, glycine, hexadecanoic acid, l-threonine, butanoic acid, malic acid, l-proline and propanoic acid levels increased significantly (*p <* 0.05) in the BD group, the levels of d-fructose in the BD group decreased significantly (*p <* 0.05). The relative concentrations of 12 endogenous metabolites were also significantly affected by the ASL, ASM, and ASH treatments. Notably, most of the altered BD-related metabolites were restored to normal state after ASM administration.

**Conclusion:** AS can promote hematopoietic activities, inhibit production of reactive oxygen species, regulate energy metabolism, increase antiapoptosis, and potentially contribute to the blood enrichment effects of AS against APH- and CTX-induced BD mice.

## Introduction

Anaemia is a common disease characterized by a decrease in haemoglobin (HGB). Anaemia frequently occurs because of fatigue, pressure, and radiation. Different types of this disease include blood loss anaemia, aplastic anaemia, sickle-cell anaemia, and iron-deficiency anaemia (Gupta 2014). The theory of Traditional Chinese Medicine (TCM) states that blood loss anaemia is similar to blood deficiency (BD) in TCM (Shi et al. 2014), such as the condition of postoperative and postpartum women with chronic bleeding, excessive menstruation, prolonged menstrual periods or uterine bleeding.

*Angelica sinensis* (Oliv.) Diels (Umbelliferae) (AS) is a well-known TCM that enriches and regulates the blood. AS is a popular herb commonly used in clinics for the treatment of BD syndrome in China. AS mainly consists of polysaccharides and different bioactivities, such as haematopoietic (Yang et al. 2009; Zhao et al. 2012), immunomodulatory (Ko & Cho 2003), antitumor (Cao et al. 2006), antioxidant (Ai et al. 2013) and antiulcer (Ye et al. 2003) effects. Several researchers have investigated the distribution of AS components, including phthalides, these components exhibit antifungal, antibacterial, anti-inflammatory, smooth-muscle relaxant, and vasodilatory properties (Chao et al. 2010; Su et al. 2011). AS is also a basic component of many Chinese drugs used for BD, such as DangguiBuxuetang (Shi et al. 2014) and Siwutang (Su et al. 2008). Although, AS has been applied clinically in animal models, the mechanism of AS blood enrichment effects remains unclear.

Proteomics and metabonomics are two well-established ‘-omic’ techniques in the post-genomic era. Proteomics is the study of large-scale proteins in an organism encoded by its genome. It is a powerful tool and has been widely used to elucidate protein profile changes in response to drug treatment, as well as to identify disease-relevant biomarkers (Hsiao et al. 2012). Metabonomics focuses on a global profile of metabolites with low molecular weight (1000 Da), which are the end products of metabolisms in biofluids, tissues and even whole organisms (Sheridan et al. 2012). This approach is consistent with the integrity and systemic feature of TCM, which is increasingly utilized as a versatile tool for assessing the therapeutic effects and toxicities of many herbs used in TCM, such as *Poriacocos* (Schw.) Wolf (Polypores) (Zhao et al. 2013), Xin-Ke-Shu (Liu et al. 2014) and Wutou decoction (Qi et al. 2014), as well as *Pinelliaternata* (Thunb.) Berit (Araceae) (Zhang et al. 2013) and ChaihuShuGanSan (Su et al. 2011). Proteomics is also applied to assess the therapeutic effects and toxicities of many herbs used in TCM (Lao et al. 2014), including YinChenHaoTang (Sun et al. 2013), Shuanglong Formula (Fan et al. 2010), bear bile powder (Wang et al. 2013), *Salvia miltiorrhiza* Bge. (Lamiaceae), and *Panax notoginseng* (Burk), F. H. Chen (Araliaceae) (Yue et al. 2012). Both proteomics and metabonomics are frequently used as versatile tools in such assessment function (Buriani et al. 2012). Any omic platform provides a limited window in the biological activity of an isolated system (Anonymous) (Rantalainen et al. 2006). Many examples of the use of parallel omic platforms for studying cellular and complex organism systems exist, including yeast (Gygi et al. 1999), plants, and mammals (Rantalainen et al. 2006). These reports indicate that a combination of proteomics and metabonomics can validate and complement each other when testing the pharmacological effects of TCM, especially in a complex system.

In the present study, the BD model was induced by a combination of acetyl phenylhydrazine (APH) and cyclophosphamide (CTX), and this model was consistent with the inner environment of blood deficiency (Jia et al. 2011). Two ‘-omic’ approaches, namely, traditional 2D gel electrophoresis (2-DE)-based proteomics and gas chromatography–mass spectrometry (GC-MS)-based metabonomics, were used to investigate the effects and blood enrichment mechanisms of AS by inducing the BD model in mice by using APH and CTX.

## Materials and methods

### HPLC chromatograms of AS and its constituent herbs

#### Solvents and chemicals

Analytical grade methanol (Basifu Chemical Reagent Co. Ltd, Tianjin, China) and formic acid (Laiyang Chemical Reagent Co. Ltd, Shandong, China) were used for sample preparation. HPLC grade acetonitrile (Fisher, Pittsburgh, PA), deionized water obtained from a Milli-Q water system (Millipore, Bedford, MA) and analytical grade glacial acetic acid (Basifu Chemical Reagent Co. Ltd, Tianjin, China) were used for preparation of mobile phase. Sampling part and sample source of 11 tested samples are summarized in Table 1.

**Table 1.** Origins of AS from Gansu.

Sample code	Source	Sampling part	Date of collection
S1	Jinzhongzhen, Zhangxian	Whole root	September, 2014
S2	Zhongzhaizhen, Minxian	Whole root	September, 2014
S3	Xizhaizhen, Minxian	Whole root	September, 2014
S4	Mazichuanxiang, Minxian	Whole root	September, 2014
S5	Tianjiahezhen, Weiyuan	Whole root	September, 2014
S6	Wuzhuzhen, Weiyuan	Whole root	September, 2014
S7	Huichuanzhen, Minxian	Whole root	September, 2014
S8	Dacaotanxiang, Zhangxian	Whole root	September, 2014
S9	Shichuanxiang, Zhangxian	Whole root	September, 2014
S10	Shilixiang, Minxian	Whole root	September, 2014
S11	Minxian market of Chinese herb	Whole root	September, 2014

#### Reference compounds preparation

Ferulic acid was purchased from the Institute for the Control of Pharmaceutical and Biological Products of China (Beijing, china). Ferulic acid (4.8 mg) was dissolved and diluted to 100 mL with methanol.

#### Sample preparation

An accurately weighed sample powder of 0.5 g was transferred to 50 mL Erlenmeyer flask and 25 mL methanol–formic acid (95:5) was added. The weight of vial was record and the Erlenmeyer flask was sealed and sonicated for 60 min. the original solvent weight was restored. The extract was filtered through a 0.45 μm membrane filter. An aliquot of 10 mL solution was injected for HPLC analysis.

#### HPLC conditions

Samples chromatograms were acquired using an Agilent/HP 1100 series HPLC-UV system consisting of a vacuum degasser, binary pump, auto sampler, thermostatted column compartment and binary wavelength detector (Agilent, Palo Alto, CA), and fitted with a and fitted with a ODS -C_18_ reversed-phase column (5 μm, 250 mm × 4.6 mm).

#### AS preparation

AS root was purchased from Minxian County, Gansu Province, China and authenticated by Dr. Yanming Wei (College of Veterinary Medicine, Gansu Agriculture University, Lanzhou, China). Voucher specimens are stored in the herbarium centre of Gansu Agriculture University (Root of AS, AS201409001). AS samples were pulverized into powder and passed through a 40-mesh sieve. The AS samples (400 g) were weighed and immersed in 1200 mL of distilled water for 1 h, then extracted in boiling water for another 1 h. Supernatant was obtained after cooling and filtering. The residues were decocted with half amount of distilled water and boiled for 0.5 h. The supernatant was then removed. Two decoctions were mixed and filtrated, then concentrated with rotary evaporators to 300 mL. The samples were transferred into a volumetric flask, and distilled water was added until the volume reached 400 mL. Each sample contained 1 g/mL of rude drugs. The samples were stored at 4 °C for future use.

#### Grouping and administration

Kunming male mice (18 g to 22 g) were supplied by the Experimental Animal Center of Gansu Agricultural University (Lanzhou, China). Room temperature was regulated at 20 °C to 30 °C. A 12 h light/dark cycle was set, and the mice were fed standard diet with free access to water. The mice were allowed to acclimatize for 2 d prior to dosing in the metabolic cages. After acclimatization, the mice were randomly divided into five groups with eight mice in each group as follows: control group, BD group, high-dose group of AS decoction (ASH), medium-dose group of AS decoction (ASM), and low-dose group of AS decoction (ASL). ASH, ASM and ASL were given 20, 10 and 5 g/kg. The animal dose of ASH, ASM and ASL extract was extrapolated from the human daily dose, using the body surface area normalization method (Xu et al. 2001), so animal drug dose calculated on body surface area of conversion factor: clinical dosage ×0.018/200 × 1000 × clinical equivalent amount of multiples) body mass of AS decoction with 0.2 mL, respectively, by oral gavage once every day continuously for seven days from the day 1. At the same time, mice in BD and control groups were given an equal volume of normal saline (NS). BD, ASH, ASM and ASL were hypodermically injected with 2% APH saline solution on days 1 and 4 at dose of 20 mg/kg and 40 mg/kg, respectively; 2 h after the hypodermic injection with 2% APH saline solution on day 4, the mice were intraperitoneally injected with CTX saline solution on day 4, 5, 6 and 7 at a dose of 40 mg/kg. Thus, the blood deficiency model was created. Blood sample of mice was collected by posterior orbital venous plexus approach on day 7 and their livers were harvested. The red blood cell (RBC), white blood cell (WBC), HGB, and hematocrit (HCT) levels were also examined using an auto-hemocytome by Mindray BC-5300 automatic blood analyzer. The harvested livers were flash frozen in liquid nitrogen and stored at −80 °C for further use.

Animal welfare and experimental procedures were performed in accordance with the Guide for the Care and Use of Laboratory Animals (Ministry of Science and Technology of China, 2006) and approved by the Animal Ethics Committee of Gansu Agricultural University.

### Proteomics analysis

#### Sample preparation for proteomics

For each treatment, livers (0.1 g) were manually homogenized in 1 mL of lysis buffer, a solution that contained 7 M urea, 2 M thiourea, 4% (w/v) CHAPS, and 40 mM DTT. Pellets were suspended in 10% TCA with 20 mM DTT. Proteins were precipitated on ice for 1 h. Supernatant was discarded, and the protein pellets were washed thrice with cold acetone that contained 20 mM DTT after centrifugation at 12,000 *g* for 15 min at 4 °C. Finally, the pellets were dried and resuspended in 350 μL of lysis buffer together with 0.5% (v/v) IPG buffer (pH 3 to pH 10 NL) and 0.001% (w/v) bromophenol blue. Protein concentrations were determined by Bradford method.

#### Two-dimensional electrophoresis, image acquisition, and image analysis

The first dimension showed isoelectric focusing (IEF) with IPGs. The protein samples (340 μL that contained approximately 900 μg of proteins) were loaded onto 18 cm immobiline dry strips (pH 3 to pH 10 NL; GE Healthcare) and applied by in-gel rehydration in the re swelling tray. The strips were rehydrated overnight at room temperature. IEF was performed the following day using IPGphor II (GEhealthcare) at approximately 20 °C under the following conditions: 200 V for 1 h, 500 V for 1 h, 1000 V for 2 h, gradient from 1000 V to 8000 V within 1 h, and 8000 V for 5 h. Prior to the second dimension, the IPG strips were subjected to equilibration in a buffer that contained 6 M urea, 2% (w/v) SDS, 50 mMTris-HCl (pH 8.8), 30% (w/v) glycerol, and 1% (w/v) DTT for 15 min. This process was followed by a 15 min equilibration in the same buffer that contained 2.5% (w/v) iodoacetamide instead of DTT. The 2D-E was performed on 12% SDS-polyacrylamide gels at 80 V for 4 h, 100 V for 4 h, and 120 V for 2 h. The gels were stained using an MS-compatible silver staining method, in which glutaraldehyde was omitted from the sensitizer. Three independent experiments were performed. Gel image acquisition was performed using Image Scanner III (GE Healthcare). All images were imported and analyzed using ImageMaster 2D Platinum 7.0 (GE Healthcare) in accordance with the user manual instructions. Spots were automatically detected in each gel based on the detection parameters, and a manual edition was performed as necessary. The relative volume of a protein spot (% vol) was calculated and used for comparison. Using three biological replicate analyses, only statistically significant (*p*-value <0.05) protein spots that changed ≥1.5-fold were considered differentially expressed proteins.

#### Protein identification by MALDI-TOF/TOF MS and database searches

Selected gel spots were manually excised and placed in micro-centrifuge tubes for in-gel digestion. First, gel pieces were washed twice with Milli-Q water followed by dehydration with acetonitrile. Second, each gel piece was reswelled in a 5 μL digestion buffer that contained 25 mM NH_4_HCO_3_ and 12.5 ng/μL porcine trypsin (Promega) on ice for 45 min. Excess trypsin was removed, and 10 μL of the same buffer without trypsin was added. Finally, proteins were digested overnight with trypsin at 37 °C.

The digestion solution was directly deposited on an Anchor Chip plate (Bruker Daltonics) at 5 μL per spot, followed by 1 μL of matrix solution of 0.4 mg/mL HCCA in 70% acetonitrile. Finally, 1 μL of TFA was detected to remove the remaining salt ions. Tandem mass spectrometry was performed using an Ultraflex III MALDI TOF/TOF mass spectrometer (Bruker Daltonics). The peak lists of MS and MS/MS spectra were combined using BioTools software 3.1 (Bruker Daltonics) and sent to a Mascot search engine (Matrix Science) against our custom-built Expressed Sequence Tag (EST) database (unpublished data), NCBInr database, or EST others database. Only the matches with significant scores (*p* < 0.05) were accepted. Based on the UniProt Knowledgebase (http://www.uniprot.org/), identified proteins were submitted to the Kyoto Encyclopedia of Genes and Genomes (KEGG) and classified according to the KEGG pathway maps (http://www.genome.jp/kegg/pathway.html). Some of the proteins cannot be assigned on the basis of the KEGG pathway maps. Thus, these proteins were classified in accordance with the biological processes of Gene Ontology (http://www.geneontology.org/).

#### Sample extraction and derivatization for metabonomics

The liver samples were thawed at room temperature prior to analysis. Approximately 900 μL of methanol was then added into 100 μL of the sample to precipitate the protein. The mixture was shaken vigorously for 60 s. A 20 mL docosane solution (0.1 mg/mL) was added into the mixed sample as internal standard. The mixture was then ultrasonically extracted for 10 min, followed by centrifugation (12,000 *g*) for 10 min. Approximately 400 μL of supernatant was transferred to the GC vial and evaporated to dryness under a stream of nitrogen gas. The sample metabolites were chemically derived using a combination of methoxymation and silylation. A 100 μL aliquot of methoxyamine pyridine solution (15 μg/μL) was added to the vial. Methoxymation was performed at 70 °C for 1 h. Approximately 180 μL of MSTFA with 1% TMCS as catalyst was then added to the vial. After silylation at 70 °C for 1 h, the sample solution was transferred to the GC micro-vial for GC-MS analysis after filtration. Derivation was conducted as previously reported (Huang et al. 2008).

### Metabolite analysis

#### Chromatography

GC-MS was used to analyze the liver tissue samples. Chromatographic analysis was performed in an Agilent 6890N/5973N series GC-MS (Agilent Technologies, Santa Clara, CA) equipped with an OV-1701 capillary column (30 m × 0. 5 μm × 0.25 mm).

#### Analytical procedure

The initial temperature (85 °C) was held constant for 3 min then increased to 280 °C at a rate of 10 °C/min. All samples were injected in split mode. The injection temperature was 270 °C. The mass spectrometer was operated in EI mode (positive ion, 70 eV), and the quadrupole was 150 °C. Mass spectra were acquired in full scan mode with repetitive scanning from 60 to 600 m/z in 1 s. The ion source temperature was 230 °C.

### Statistical analysis

Biochemical parameters were expressed as mean ± standard deviation. All the statistical analyses were conducted using SPSS for Windows (version 13.0).

Raw data acquired from GC-MS were converted by Masshunter B.0 6.00 software (Agilent Technologies, Santa Clara, CA) for peak deconvolution and detection using the ‘Find by Chromatographic Deconvolution’ algorithm. The detected components with abundance of <5000 counts and relative abundance of <1% were removed. The convoluted data were subsequently imported into the Mass Profile Professional software (Agilent Technologies, Santa Clara, CA) for peak alignment, filtering, baseline correction, and area calculation. Molecular features that existed in at least 80% of the samples in either group were retained. Abundance values were normalized to that of the internal standard and log transformed. Finally, a spreadsheet that contained relative peak intensities, compound name and sample information was exported for succeeding analyses.

Pattern recognition based on partial least squares-discriminant analysis (PLS-DA) was accomplished using SIMCA-P V11.0 (Umetrics, Sweden) after mean centering and auto-scaling for equal metabolite measurement.

Potential markers of interest were extracted from loading plots constructed after PLS-DA, and markers were selected based on their contribution to the variation and correlation within the data set. The metabolites in the GC-MS spectra were identified by comparing the experimental mass spectra with those in the National Institute of Standards and Technology mass spectra library. Metabolic pathway analysis (MetPA) was performed using Metaboanalyst (http://www.metaboanalyst.ca) to reveal the disturbed metabolism.

## Results

### Chromatograms of as extract and chemical constituents identified

**Figure 1.**
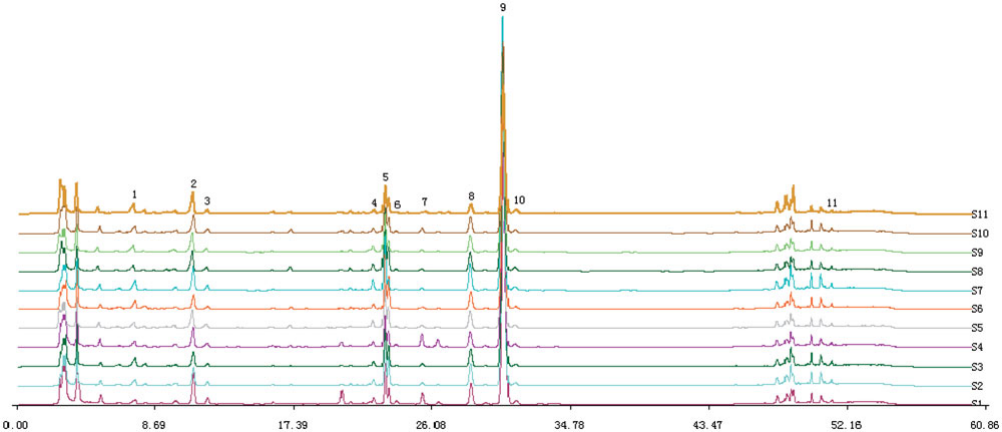
HPLC fingerprint graphics of AS. Analytical column: ODS-C18, 4.6 mm ×25 cm,5 μm; injected sample volume: 10 μL; mobile phase: 1.0% acetic acid in water (A) and acetonitrile (B) using a gradient program of 20–30% (B) in 0–10 min, 30–49% (B) in 1 0–20 min, 49% (B) in 2 0–40 min; 49–100% (B) in 40–50 min;100–20% (B) in 5 0–60 min; flow rate: 1 mL·min- 1; temperature: 25 °C; UV detection: 280 nm. (1) ferulic acid; (2) senkyunolide I; (3) senkyunolide H; (4) unkown compounds; (5) coniferyl ferulate; (6) senkyunolide A; (7) butylphthalide; (8) E-ligustilide; (9) *Z*-ligustilide; (10) *Z*-butylidenephthalide; (11) levistolide A.

The HPLC chromatograms of AS and 11 herbs are shown in Figure 1, of which 11 characteristic peaks were assigned by comparing their retention time with those of the reference compounds, the identified results are shown in Figure 1.

### General and haemogram observation of mice

Normal (control) mice were strong and vigorous and presented brilliant pink eyes, clean pink moist nose and lips, round pink tail, and straight back and loin. Their fur was luxuriant and lustrous. The animals appeared exhausted, sluggish, asthmatic, and somnolent 3 d after APH administration. Their bodies were curled into a ball with raised facial hair, and their ears and eyes were pale and cool. BD signs in the mice matched the same description of BD symptoms.

The peripheral blood indexes in the control, BD, ASH, ASM and ASL mice are shown in Table 2, including the WBC, RBC, and HGB values, which were the main diagnostic criteria of the BD syndrome. The results showed that the RBC, HGB, WBC and HCT in the BD mice decreased significantly (*p* < 0.05) unlike in normal mice, indicating that the BD model was successfully induced.

**Table 2.** The impact on peripheral blood of AS decoction.

	*N*	RBC/×10^12^	WBC/×10^9^	Hb/g·L^−1^	PLT/×10^9^
Control	8	9.36 ± 1.39	7.18 ± 1.65	142.6 ± 18.19	719.0 ± 306.47
BD	8	6.1 ± 0.37*	4.24 ± 1.44*	110.0 ± 6.16*	518.8 ± 226.04*
ASH	8	8.13 ± 0.39^▴^	4.72 ± 3.32^▴^	128.6 ± 3.36^▴^	653.0 ± 260.47^▴^
ASM	8	8.42 ± 0.31^▴^	5.02 ± 1.2^▴^	130.4 ± 13.89^▴^	685.4 ± 220.29^▴^
ASL	8	7.8 ± 1.31^▴^	6.16 ± 1.71^▴^	120.2 ± 7.63^▴^	641.2 ± 303.85^▴^

Compared with BD group, *p < 0.05.

Compared with control group, ^▴^*p* < 0.05.

### Comparative proteome of control, BD, ASH, ASM and ASL groups

**Figure 2.**
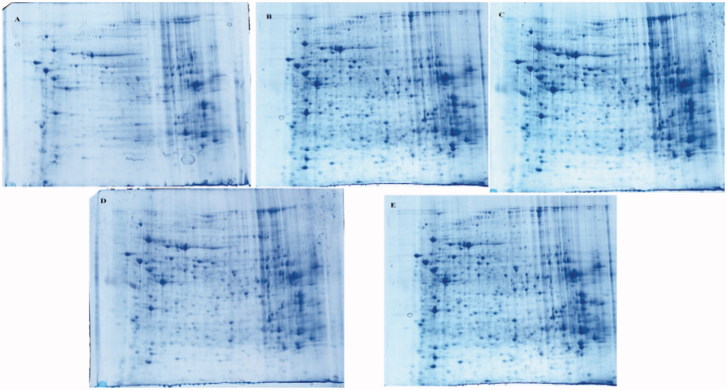
Differential effect of AS treatment on BD as visualized by 2D PAGE. Liver were extracted and analyzed by 2D PAGE (first dimension, 18 cm, pH 4–7, nonlinear gradient of IPG strips; second dimension, 12.5% SDS-PAGE) and visualized by silver staining. (A) control; B BD; C ASM; D ASL;E ASH;.

**Figure 3.**
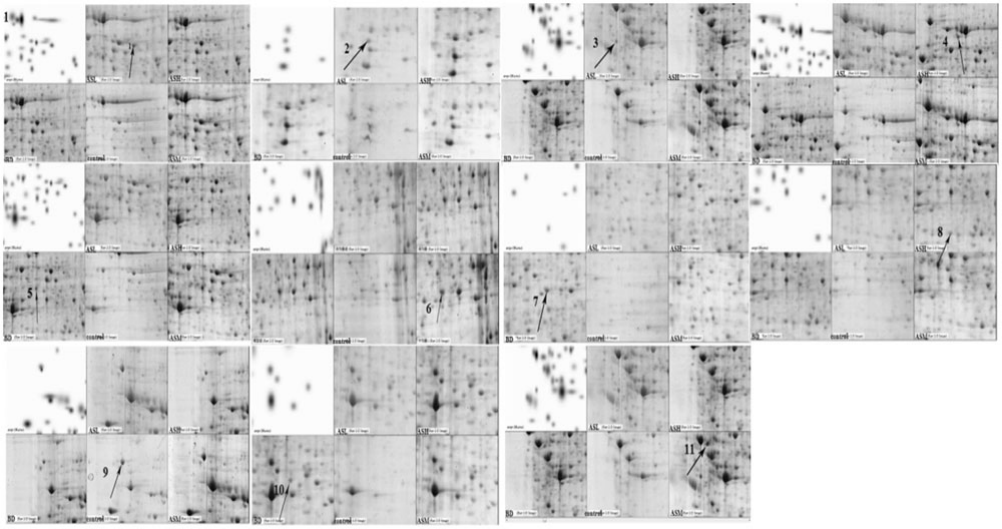
Protein expression profiles of mouse liver tissues of control, BD, ASL, ASM, ASH group. The protein expression profiles were examined using 2-DE system which were demonstrated by representative gel images. To ensure reproducible results for each protein sample, duplicate or triplicate electrophoreses were performed. Differentially expressed spots between control group and BD group were found by comparing the spot intensities using PD-Quest software. Differentially expressed spots were indicated in the images by arrows. There were11 differentially expressed spots that could be identified from the AS extract, respectively, compared with the DMSO control. The numbered protein spots were identified by MALDI-TOF/TOF mass spectrometry. The corresponding protein spot identities are shown in Table 2.

2D-E combined with silver staining was performed to determine the difference in the proteomes of the control, BD, ASH, ASM and ASL groups. A total of 11 differentially expressed protein spots were found in the BD group (Figures 2 and 3).

### Mass-spectrum identification and bioinformatics analysis of differentially expressed proteins

All differentially expressed proteins were identified using a MALDI-TOF/TOF mass spectrometer. Nine of the eleven differentially expressed proteins were successfully identified. The identified proteins include carbonic anhydrase, glyceraldehyde-3-phosphate dehydrogenase (GAPDH), catalase, fibrinogen, glutathione *S*-transferase Pi (GSTP), arginase, heat shock protein 90 (HSP90), carboxylesterase, and haeme-binding protein.

Unlike in the control group, the HSP90 and arginase levels increased significantly (*p* < 0.05) in the BD group, but the levels of carbonic anhydrase, GAPDH, catalase, fibrinogen, GSTP, carboxylesterase and haeme-binding protein in the BD group decreased significantly (*p* < 0.05). Unlike the levels in the BD group, the levels of these biomarkers were regulated to a normal state near the control group in the ASM group. The results are listed in Table 3.

**Table 3.** AS influence on liver proteins of BD mice.

no	Protein name	Control	BD	ASH	ASM	ASL
1	Heat shock protein 90	0.51 ± 0.015	0.83 ± 0.006*	0.73 ± 0.018	0.53 ± 0.012^▴^	0.60 ± 0.007
2	Carbonic anhydrase	0.13 ± 0.011	0.04 ± 0.008*	0.06 ± 0.015	0.09 ± 0.012^▴^	0.04 ± 0.022
3	Glyceraldehyde-3-phosphate dehydrogenase	0.43 ± 0.008	0.05 ± 0.023*	0.32 ± 0.007	0.42 ± 0.019^▴^	0.29 ± 0.013
4	Catalase	0.33 ± 0.016	0.11 ± 0.019*	0.26 ± 0.027	0.32 ± 0.016^▴^	0.14 ± 0.031
5	Fibrinogen	0.53 ± 0.023	0.28 ± 0.031*	0.43 ± 0.018	0.51 ± 0.032^▴^	0.37 ± 0.041
6	Heme-binding protein	0.46 ± 0.032	0.16 ± 0.033*	0.34 ± 0.051	0.41 ± 0.016^▴^	0.28 ± 0.024
7	Glutathione *S*-transferase Pi	0.62 ± 0.025	0.33 ± 0.034*	0.46 ± 0.048	0.58 ± 0.037^▴^	0.37 ± 0.018
8	Arginase	0.18 ± 0.018	0.39 ± 0.023*	0.22 ± 0.052	0.19 ± 0.043^▴^	0.20 ± 0.017
9	Carboxylesterase	0.37 ± 0.041	0.18 ± 0.024*	0.3 1 ± 0.036	0.33 ± 0.047^▴^	0.28 ± 0.026

Compared with BD group, *p < 0.05.

Compared with control group, ^▴^*p* < 0.05.

### Comparative metabonomics in control, BD, ASH, ASM and ASL groups

**Figure 4.**
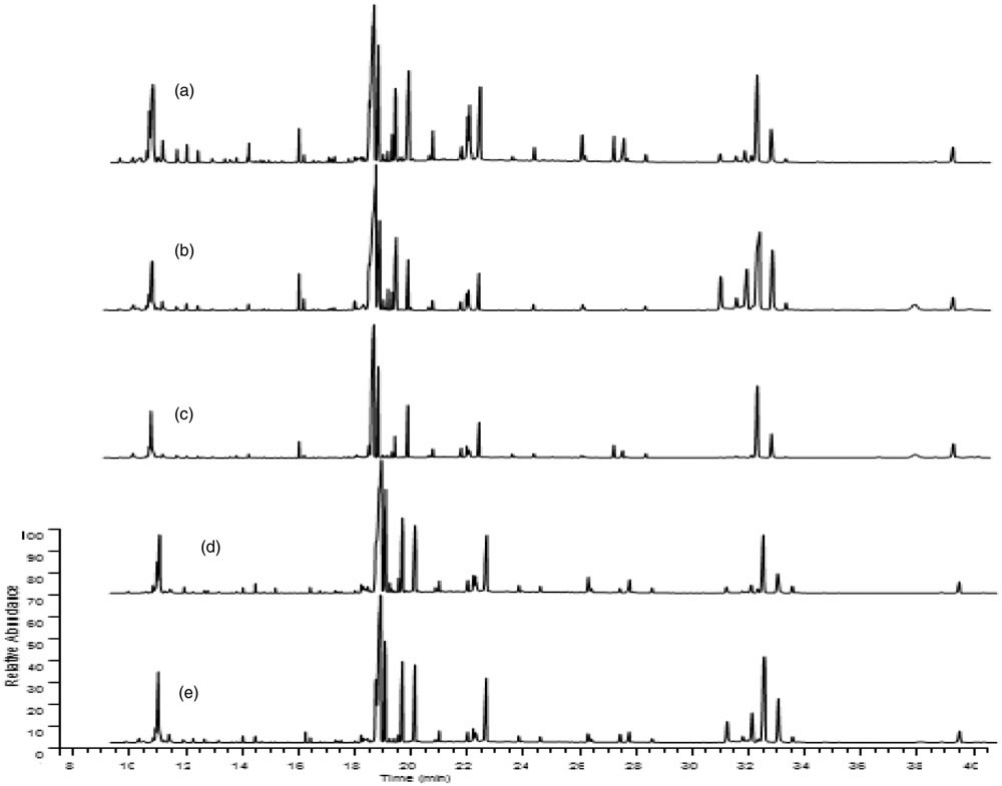
GC-MS TIC of the liver tissue homogenate (a) Control; (b) BD; (c) ASH; (d) ASM; (e) ASL.

**Figure 5.**
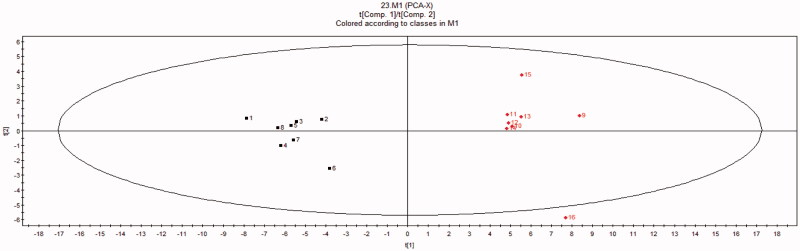
PCA score plots of liver tissue homogenate from the BD (1-8) and control (9-16) groups. Evident separated clustering of liver injury and the control groups are noted.

**Figure 6.**
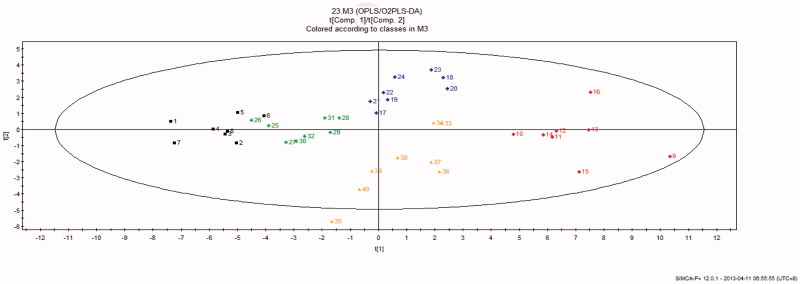
PLS-DA score plots of liver tissue homogenates Modelling Diagnostic: *R*^2^*X* = 0.311, *R*^2^*Y* = 0.930, *Q* = 0.78; 1–8, control; 9–16, BD; 17–24, ASH; 25–32, ASM; 33–40, ASL.

Representative GC-MS total ion chromatograms of the liver metabolite profiles are shown in Figure 4. A total of 23 compounds were identified in liver tissue homogenates (Table 4). The peak areas of all metabolites were subjected to PCA and PLS-DA. Score plots of PCA measured the separation between the BD and control samples (Figure 5). The PLS-DA score plots (Figure 6) of the livers in all groups showed that all the samples were perfectly separated, and the samples in every group were well clustered. This scenario indicates that the physiological metabolism of BD mice was interrupted, demonstrating that the BD model is effective in the aspect of physiological metabolites. The abnormal metabolic profiling of the BD mice was also clearly regulated by providing AS to these mice. Among all the AS groups situated near the control group, ASM was the nearest to the control group. This finding indicates that ASM demonstrated the optimal moderating effects on the BD mice.

**Table 4.** Endogenous metabolites in the liver tissue homogenate.

Retention time (min)	Match	Probability	Endogenous metabolites
9.55	910	85.4	L-Valine
10.02	901	86.5	Urea
11.08	897	83.9	Glycine
11.94	921	92.6	Lactic acid
12.03	900	81.2	Oxalic acid
12.33	913	84.2	L-Threonine
12.51	938	98.6	Butanoic acid
12.84	891	83.1	L-Alanine
13.47	901	95.6	Aminomalonic acid
13.70	925	85.1	Malic acid
14.14	932	88.7	L-Proline
15.04	870	80.5	2-Hydroxybutanedioic acid
16.10	908	83.8	D-Fructose
17.00	911	74.4	Phosphoric acid
17.19	917	81.4	Succinic acid
21.90	896	83.4	9,12-Octadecadienoic acid
19.80	893	90.3	Hexadecanoic acid
20.68	913	80.7	myo in ital
21.97	934	84.2	Oleic acid
22.33	925	83.5	Octadecanoic acid
24.28	943	86.3	Arachidonic acid
25.99	928	85.9	Uridine
39.16	913	82.6	Cholesterol

Potential biomarker screening was performed based on the VIP value (VIP >1.0), and a significant test using the PLS-DA model was conducted between the BD and control groups. The variables significantly contributed to the PLS-DA model only when they satisfied the conditions of VIP >1.0 and *p* < 0.05. The relative concentrations of 12 endogenous metabolites (Table 5) were also significantly affected by the ASL, ASM, and ASH treatments. Notably, most of the altered BD-related metabolites were restored to normal state after ASM administration.

**Table 5.** AS influence on liver metabolites of BD mice.

Metabolite	Retention time	VIP value	Control	BD	ASH	ASM	ASL
L-Alanine	13.84	1.1689	0.16 ± 0.13	0.36 ± 0.12	0.24 ± 0.16	0.20 ± 0.23^a^	0.27 ± 0.06^▴^
Arachidonic acid	24.28	1.1602	0.11 ± 0.13	0.40 ± 0.08	0.23 ± 0.16^a^	0.19 ± 0.23^b^	0.28 ± 0.21^a^^▴^
L-Valine	9.55	1.1530	0.11 ± 0.27	0.18 ± 0.13	0.14 ± 0.32	0.12 ± 0.40^a^	0.16 ± 0.39^▴^
Octadecanoic acid	22.33	1.1443	0.20 ± 0.05	0.32 ± 0.03	0.27 ± 0.05	0.25 ± 0.11^a^	0.28 ± 0.04^▴^
Glycine	11.08	1.1325	0.30 ± 0.13	0.56 ± 0.20	0.41 ± 0.22	0.34 ± 0.20^b^	0.46 ± 0.21^▴^
Hexadecanoic acid	19.80	1.1192	0.20 ± 0.04	0.31 ± 0.12	0.26 ± 0.02	0.20 ± 0.09^b^	0.27 ± 0.05
L-Threonine	12.33	1.0990	0.21 ± 0.08	0.33 ± 0.04	0.28 ± 0.09	0.25 ± 0.14^a^	0.30 ± 0.12^▴^
Butanoic acid	12.51	1.0933	0.38 ± 0.09	0.53 ± 0.10	0.42 ± 0.06	0.41 ± 0.08^a^	0.43 ± 0.11
Malic acid	13.70	1.0723	0.85 ± 0.25	2.04 ± 0.01	1.32 ± 0.20^a^	1.06 ± 0.15^a^	1.55 ± 0.06^▴^
D-Fructose	19.10	1.0613	0.32 ± 0.09	0.13 ± 0.09	0.21 ± 0.15	0.26 ± 0.14^a^	0.16 ± 0.88^▴^
L-Proline	14.14	1.0493	0.28 ± 0.10	0.46 ± 0.110	0.35 ± 0.05^▴^	0.31 ± 0.05^b^^▴^	0.34 ± 0.24^▴^
Propanoic acid	11.94	1.0162	0.18 ± 0.11	0.34 ± 0.12	0.25 ± 0.05^▴^	0.24 ± 0.14^a^^▴^	0.26 ± 0.08^▴^

Compared with BD group, ^a^*p <* 0.05, ^b^*p <* 0.01.

Compared with control group, ^▴^*p <* 0.05.

### Metabolic pathway analysis

**Figure 7.**
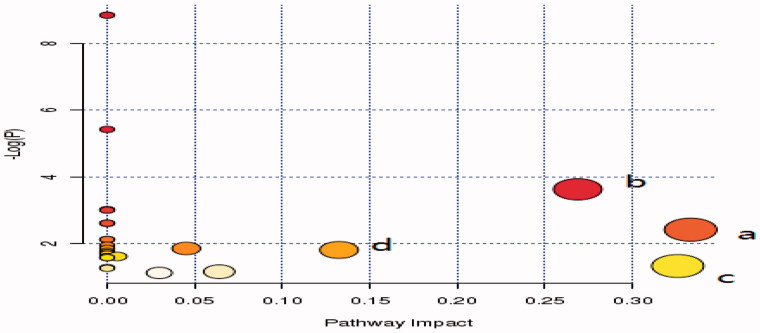
Summary of Pathway Analysis with MetPA; (a) Valine, leucine and isoleucine biosynthesis, (b) Glycine, serine and threonine metabolism, (c) Arachidonic acid metabolism, (d) Fructose and mannose metabolism.

The metabolic pathway analysis with MetPA (www.metaboanalyst.ca) revealed that the identified biomarkers involved in valine, leucine, and isoleucine biosynthesis; arachidonic acid (AA) metabolism; glycine, serine, and threonine metabolism; and fructose and mannose metabolism were 0.33, 0.33, 0.27, and 0.14, respectively. The pathway effect value >0.1 calculated from the pathway topology analysis was excluded as a potential target pathway (Figure 7).

## Discussion

### Proteins and metabolites involved in haematopoietic activities

**Figure 8.**
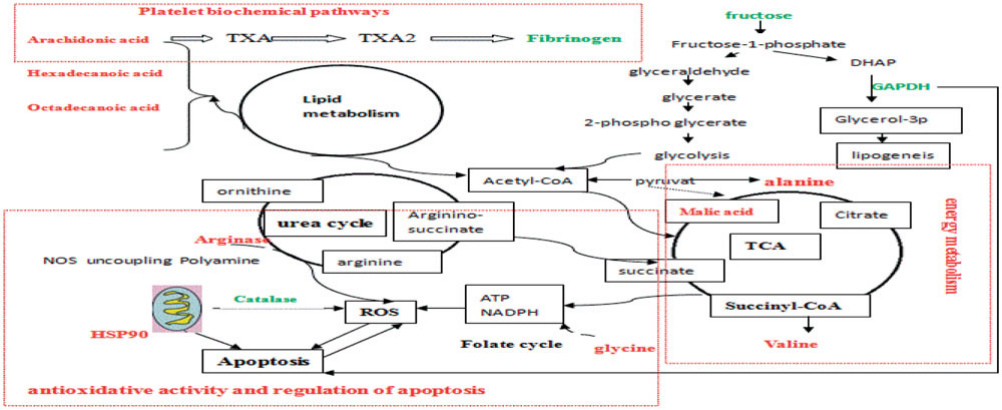
The summarized change at proteomic and metabonomic levels in BD mice liver. Metabolites and proteins in red and green represent an increasing and decreasing concentration in the APH and CTX that interfered, respectively.

Haeme-binding proteins can bind free porphyrinogens possibly present in the cell to facilitate the removal of these potentially toxic compounds. Binds exhibit high affinity to a haeme or porphyrin molecule. These proteins bind metalloporphyrins, free porphyrins, and *N*-methylprotoporphyrin with similar affinities (Jacob Blackmon et al. 2002). Heme-binding proteins play an important role in all BD phases. Fibrinogen is involved in both primary and secondary hemostasis; it plays an important role in platelet aggregation and establishment of a fibrin network. Recent evidence suggests that high levels of fibrinogen act as antithrombin, decrease the endogenous thrombin potential, and compromise clot stability, particularly following a low tissue factor stimulus (Sørensen et al. 2011). Decreased levels of the haeme-binding protein and fibrinogen were observed in the BD mice. Haeme-binding proteins and fibrinogen were up-regulated in BD by AS, which can likely promote haematopoiesis by increasing the haeme-binding protein and fibrinogen levels. AA plays an important role in regulating lipid metabolism, biological processes, and immune responses (Das 1990). Different haemopoietic growth factors clearly mediate their actions by modulating AA metabolism, which is necessary for G protein beta, gamma-subunit activation, Ptd Ins (4, 5) P_2_ pathway (i.e., diacylglycerol, which is the second messenger derived through the hydrolysis of Ptd Ins 4, 5 Pz, mainly containing AA) and influencing the cyclic AMP and cyclic GMP levels in the cells (Das 1990). APH and CTX that interfered with AA metabolism can lead to agranulocytosis and anemia possibly suppressing haematopoiesis in the present study. The level of AA decreased in AS, which showed that AS recovered from AA metabolism may decrease hematopoietic damage. AA metabolic biomarkers were also confirmed in the platelet biochemical pathways by related fibrinogen (Figure 8).

### Proteins and metabolites involved in energy metabolism

Alanine, an important participant and regulator in glucose, glycine, serine, and urea cycle metabolism, increased in the model group and returned to normal level after oral administration of 10 and 20 mg/kg AS for 7 d. Normal alanine levels are acceptable in the normal energy metabolism of the body.

### Proteins and metabolites involved in antioxidative activity

Reactive oxygen species (ROS) plays an important role in the APH and CTX phases. APH, a strong oxidant, induces a slow progressive and oxidative damaging effect on RBC, resulting in haemolytic anemia (Roth et al. 1979). CTX, a chemotherapeutic agent, can deplete hematopoietic stem cells in the marrow and circulating peripheral blood cells, leading to hematopoietic suppression and immunodeficiency injuries (Tiev et al. 2014); however, excessive ROS generation can lead to changes in cell functions and eventually to cell death. Catalase and GSTP, which are up-regulated in the liver by AS, decreased in the BD group. Catalase is an important enzyme protecting the cell from oxidative damage by ROS (Golenia et al. 2014). Catalase activity can be implicated in the defence against excessive oxidative stress, not only in erythrocytes, but also in the plasma and serum of sporadic amyotrophic lateral sclerosis patients and healthy subjects (Golenia et al. 2014). GSTP promotes the conjugation of glutathione with carcinogens, as well as compounds produced by radical reactions and lipid peroxidation, which are important in chemoprevention and detoxification (Tew et al. 2011). Nevertheless, the amount of hydrogen peroxide in the liver after AS treatment was substantially decreased, indicating the presence of antioxidants in cells after drug treatment. Our results collectively revealed that the ROS significantly decreased in the cells after AS treatment, which likely involved catalase and GSTP.

Glycine, serine and threonine metabolism affected ant oxidative activity (Amelio Cutruzzola et al. 2014). The increased glycine in the model group was observed in the current study and may participate in the ant oxidative activity during AS development.

Our studies on the BD model revealed that the urea cycle enzyme arginase increased. Excessive arginase activity decreased the l-arginine supply for nitric oxide synthase, which causes it to become uncoupled and produce superoxide and less nitric oxide. Superoxide and NO react rapidly and form the toxic oxidant peroxynitrite. Glutamate and the catabolic products of polyamine oxidation can induce more oxidative stress and DNA damage. Our results indicated that APH and CTX injuries in the BD mice models were associated with increased arginase expression/activity, which can decrease NO and increase the available ROS (Narayanan et al. 2013). Thus, we postulate that the activation of the arginase/polyamine pathway plays an important role in the BD model. The results showed that arginase activity and expression were substantially elevated in the plasma, RBCs, and platelets from sickle-cell disease patients (Morris 2014). These scenarios were similar to the analysis results obtained from our study. Arginase returned to normal level after oral administration of AS for 7 d.

The disturbance in antioxidative activity induced by AS in the liver was consistent with the metabolic biomarker (i.e., glycine) and protein biomarkers (i.e., arginase, catalase, and GSTP) (Figure 8). Our results collectively revealed that ROS significantly decreased in the liver after AS treatment, which likely involved glycine, arginase, catalase, and GSTP. Our previous research work also confirmed AS can remove oxygen-free radicals.

### Proteins and metabolites involved in apoptosis regulation

Heat-shock proteins (HSPs) are highly regulated proteins involved in normal cellular activity; HSPs are upregulated when the cell is exposed to stress, such as heat or excessive ROS production (Kregel 2002). HSPs are critical factors in mediating stress responses. Hsp90 can also prevent apoptosis by interacting with and stabilizing receptor-interacting protein-1 (an ant apoptotic protein) and the resultant activation of tumour necrosis factor-mediated nuclear factor κB (which increases the expression of genes promoting cell survival) (Lewis 2000). Moreover, Hsp90 can prevent apoptosis by binding to the apoptotic peptidase activating factor 1 (APAF-1) and inhibiting the cytochrome c-mediated formation of the APAF-1-caspase-9 apoptosome. This process prevents the activation of caspase-3 and blocks the subsequent caspase cascade leading to cell death. Hsp90 can also bind to and activate Bcl-2 that inhibits proapoptotic proteins Bax and/or Bak, thereby preventing cytochromec release from the mitochondria and suppressing apoptosis (Dejean et al. 2006). CTX and APH can increase the amount of Hsp90 expression, resulting in blockade of Hsp90 with AS. These data suggest that AS inhibits apoptosis during the BD process through HSP90 up-regulation. GAPDH, a classical glycolytic enzyme, is responsible for carbohydrate metabolism under normal conditions (Sirover 1999). However, this enzyme can be involved in many cellular processes, including DNA repair, tRNA export, membrane fusion and transport, and cytoskeletal dynamics. GAPDH also participates in cell death. However, GAPDH can mediate neuronal apoptosis after DNA damage (Sirover 2014). In the current study, GAPDH decreased in the model group, which can be involved in apoptosis regulation during AS development. GAPDH is also necessary for fructose metabolism. In this study, the findings showed that CTX and APH can decrease the fructose amount in liver tissues. Fructose preferentially suppresses apoptosis in hepatocytes after an oxidative attack by its potency to form additional NADPH for GSH regeneration through PPP stimulation, thereby significantly decreasing the cellular ROS (Frenzel et al. 2002). Notably, ASL, ASM and ASH administration gradually increased the fructose levels and reached the control levels in all cases. This observation implies that AS can inhibit apoptosis during the BD process through HSP90 upregulation.

According to the theory of traditional Chinese medicine, BD, a morbid condition of general debility which is caused by profuse bleeding and asthenia of the viscera, leading to interference of production of blood and essence; manifested as pallor, dizziness, palpitation, insomnia, numbness of extremities, small and weak pulse, etc. AS has effects of enriching blood and increasing circulation, which can improve symptoms about manifested as pallor, dizziness, palpitation, insomnia, numbness of extremities, small and weak pulse, etc. and allowed it used BD in traditional Chinese medicine.

### Relations between protein changes and metabolites

Figure 8 shows the proteomic and metabonomic responses involved in the pathways, including ROS scavenging, TCA cycle, ornithine cycle, platelet biochemical pathways, fructose metabolism, lipid metabolism, folate cycle, and energy metabolism in AS to the BD model in mice liver. The proteins associated with metabolism were involved in the Calvin cycle, fructose metabolism, lipid metabolism, urea cycle and platelet biochemical pathways (Figure 8).

## Conclusions

Using a parallel proteomic and metabonomic differential display analysis, we detected changes in proteins and metabolites in the livers of the control, BD, ASL, ASM and ASH groups. This approach facilitated further understanding of the governing blood enrichment molecular mechanisms of AS. Thirteen endogenous metabolites and nine differentially expressed proteins were tentatively identified. Four metabolic pathways contributing to BD were also determined. The metabolic and protein deviations can normalize after AS intervention. These results suggested that AS plays a pivotal role in BD treatment through down-regulation and up-regulation of endogenous metabolite and protein levels. The ASM group can recover to a healthy state better than the ASH and ASL groups. These results showed that the control mechanisms of blood enrichment involved in AS are clarified and can provide new insights into future studies, thereby contributing to a comprehensive view of AS. Our results also suggested that a combination of proteomics and metabonomics offer further insightful information about AS blood enrichment effects at molecular levels.
